# Seroprevalence and Risk Factors Associated with Hepatitis E Virus Infection in Three Species of Pet Birds in Northwest China

**DOI:** 10.1155/2014/296285

**Published:** 2014-10-30

**Authors:** Wei Cong, Qing-Feng Meng, Xiao-Feng Shan, Wu-Wen Sun, Si-Yuan Qin, Xiao-Xuan Zhang, Si-Yang Huang, Ai-Dong Qian

**Affiliations:** ^1^College of Animal Science and Technology, Jilin Agricultural University, Changchun, Jilin 130118, China; ^2^State Key Laboratory of Veterinary Etiological Biology, Lanzhou Veterinary Research Institute, Chinese Academy of Agricultural Sciences, Lanzhou, Gansu 730046, China; ^3^Jilin Entry-Exit Inspection and Quarantine Bureau, ChangChun, Jilin 130118, China

## Abstract

Hepatitis E virus (HEV), the causative agent of hepatitis E, has been reported in a wide variety of animals, including birds, but little is known of HEV infection in pet birds in northwest China. The objective of the present investigation was to examine HEV seroprevalence in three species of pet birds, namely, Eurasian siskin, Oriental skylark, and black-tailed grosbeak from Gansu. Serum samples collected from 685 pet birds from August 2011 to September 2012 were examined independently for the presence of antibodies against HEV. A total of 59 (8.31%) pet birds were tested positive for HEV antibodies by the commercially available enzyme immunoassay kits. Of these, the seroprevalence was diverse in different species pet birds; the most frequent level was 10.83% (39/360) in Eurasian siskin, followed by 6.57% (19/289) in Oriental skylark, and 2.29% (1/36) in black-tailed grosbeak. Age and collecting region of pet birds were the main risk factors associated with HEV infection. The present study firstly revealed the seroprevalence of HEV infection in three species of pet birds in northwest China, which provided the baseline data for taking comprehensive countermeasures and measures for effectively preventing and controlling HEV infection in birds.

## 1. Introduction

Hepatitis E virus (HEV), a newfound pathogen belonging to a newly recognized family of RNA viruses (Hepeviridae) [1], is the pathogenic agent of hepatitis E. In many developing countries, HEV is epidemic and endemic and it is usually transmitted via contaminated food and drinking water [2]. HEV can lead to severe hepatic disease in humans, more often with higher case fatality rates than either hepatitis A or hepatitis B virus infection. The prevalence of HEV has been reported in a wide variety of animals, including birds [3, 4].

Avian hepatitis E virus was first isolated from chickens with big liver and spleen disease or hepatitis-splenomegaly syndrome [5, 6]. Avian HEV is likely not a zoonotic virus compared to swine HEV, since it failed to infect rhesus monkeys with avian HEV [7]. Nevertheless, avian HEV alone or coinfection with other avian pathogens is clinically important in poultry industry, which can cause death and reduce egg production of chicken, resulting in significant economic losses in the poultry industry [8].

Avian HEV infection in domestic birds has been reported all over the world [4, 9, 10] as well as in China, but avian HEV infection in wild birds and pet birds has been limitedly reported [3, 11]. The objective of the present investigation was to estimate the seroprevalence of avian HEV infection in three species of pet birds, namely, Eurasian siskin (*Carduelis spinus*), Oriental skylark (*Alauda gulgula*), and black-tailed grosbeak (*Coccothraustes migratorius*) from Gansu, for the first time, which would provide “baseline” information for assessing the effectiveness of strategies for control of avian HEV infection in pet birds in China.

## 2. Materials and Methods

### 2.1. Ethics Statement

This study was approved by the Animal Ethics Committee of Lanzhou Veterinary Research Institute, Chinese Academy of Agricultural Sciences. Pet birds of various species (Eurasian siskin, Oriental skylark, and black-tailed grosbeak), from which serum samples were collected, were handled with good animal practices required by the Animal Ethics Procedures and Guidelines of the People's Republic of China. The market owners of pet birds had given permission for the collection of serum samples.

### 2.2. The Study Site

The present study was conducted in Gansu (35°50′40.9′′N 103°27′7.5′′E), northwestern China, with an area of 454,000 square kilometres, and the overwhelming majority of its land is quite high (over 1,000 meters above sea level). Gansu usually has a semiarid to arid, continental climate, with warm to hot summers and cold to very cold winters. A large proportion of the precipitation is delivered during the summer months.

### 2.3. Serum Samples

Blood samples were collected from 685 pet birds from 3 pet markets in 3 representative administrative divisions (289 from Lanzhou, 200 from Longnan, and 196 from Tianshui) in Gansu from August 2011 to September 2012. The samples were collected aimlessly from pet markets. Blood samples were collected from the wing vein of pet birds by a veterinary practitioner, taken to the laboratory, kept at room temperature for 2 h, and centrifuged at 3000 g for 10 min, and then clear serum was separated and stored at −20°C until being further tested. The information of animal husbandry practices was obtained from the pet bird market owners by questioning.

### 2.4. Serological Examination

A commercially available indirect enzyme linked immunosorbent assay (ELISA) kit (Nuoyuan Co., Ltd., Shanghai, China) was used to examine serum circulating antibodies (CAbs) to avian HEV, according to the manufacturer's instructions [11]. Negative and positive control serum samples were supplied in the kit and included in each test. Those samples with doubtful results were retested.

### 2.5. Statistical Analysis

The variation in HEV seroprevalence (*y*) of pet birds of different gender (*x*1), age group (*x*2), species (*x*3), and geographical location (*x*4) was analysed by *χ* ^2^ test using SAS version 9.1 (SAS Institute Inc., USA). In the multivariable regression analysis, each of these variables was included in the binary Logit model as an independent variable. The best model was judged by Fisher's scoring algorithm. All tests were two-sided, and values of *P* < 0.05 were considered statistically significant. Odds ratios (ORs) and their 95% confidence intervals (95% CIs) were estimated to explore the strength of the association between HEV seropositivity and the conditions investigated.

## 3. Results

In the present survey, CAbs against avian HEV were detected in 59 (8.61%, 95% confidence interval CI 6.51–10.71) of the 685 examined bird serum samples by ELISA (Table 1). Of these, the seroprevalence of avian HEV was diverse in different species pet birds; the most frequent level was 10.83% (39/360, 95% CI 7.62–14.04) in Eurasian siskin, followed by 6.57% (19/289, 95% CI 3.72–9.43) in Oriental skylark and 2.29% (1/36, 95% CI 0.00–8.15) in black-tailed grosbeak. Moreover, the seroprevalence in adult pet birds (10.37%, 56/540, 95% CI 7.80–12.94) was significantly higher than that in juvenile pet birds (2.07%, 3/145, 95% CI 0.00–4.39) (OR = 5.48, 95% CI 1.69–17.76, *P* = 0.002), but the difference between male (9.31%, 39/419, 95% CI 6.53–12.09) and female (7.52%, 20/226, 95% CI 4.35–10.69) groups was not statistically significant (OR = 0.79, 95% CI 0.45–1.39, *P* = 0.416) (Table 2).

Table 2 presents the exposure regarding species, gender, age, and collecting region associated with HEV seropositivity in pet birds based on the univariate analysis. Optimized through Fisher's scoring technique, the impacts of multiple variables on HEV were evaluated by the forward stepwise logistic regression analysis. In the final model, two variables had effects on the infectious disease, described by the following equation:

(1) $$\begin{array}{c} y=0.079x2+0.046x4+1.749. \end{array}$$

Statistical analysis suggested that the avian HEV seroprevalence in pet birds from Lanzhou (15.82%, 31/196, 95% CI 10.71–20.93) was significantly higher than that from Longnan (8.00%, 16/200, 95% CI 4.24–11.76) and Tianshui (4.15%, 12/289, 95% CI 1.85–6.45) (*P* < 0.001) (Table 2). Moreover, the seroprevalence in Eurasian siskin was significantly higher than that in black-tailed grosbeak (*P* < 0.05), but the difference between Eurasian siskin and Oriental skylark was not statistically significant (*P* = 0.059). The seroprevalence of different species from different locations ranged from 2.29% (1/36, 95% CI 0.00–8.15) (black-tailed grosbeak: Tianshui) to 15% (18/120, 95% CI 8.61–21.39) (Eurasian siskin: Lanzhou) (Table 1).

## 4. Discussion

Avian HEV infection has been isolated from the United States [6, 12], Canada [13], several countries in Europe [4, 8], Australia [5, 14], Korea [9], and China [15, 16]. However, avian HEV infection has been limitedly reported in pet birds in the world [11]. This paper represents the first report describing the seroprevalence of avian HEV from three species of pet birds (Eurasian siskin, Oriental skylark, and black-tailed grosbeak) in northwest China, and the finding from this study has significant implications for our understanding of the global epidemiology and spread of avian HEV.

Pet birds and wild birds play an important role in the public health because they may serve as natural reservoirs for many causative agents, such as Newcastle disease, avian influenza,* Chlamydia psittaci*, and others [17–20], and have been turned out to be important infection sources for a number of etiologic agents. In China, the pet birds have been raised and kept over a long period of history for companionship and entertainment. In addition, many families in China breed pet birds for commercial purposes. Previous study showed that birds are susceptible to HEV [3], which is considered an important factor influencing the economic income of the pet bird industry.

In the present study, we investigated the seroprevalence of avian HEV in three species of pet birds in Gansu. The overall avian HEV seroprevalence in pet birds was 8.61%, which was lower than that in chickens in Shandong (35.9%) [21] and Guangxi (13.35%) [22] by ELISA, in Shandong (72.2%) by RT-PCR [23], and in Korea (28%) by ELISA [9], but higher than that in parrots in Beijing and Weifang (6.43%) by the same method [11] and in pigeons in Shanghai (4.4%) [24]. The differences could be related to differences in ecological and geographical factors such as temperature, rainfall, or landscape differences. Moreover, the species of birds and the different sensitivity and veracity of the serological methods used for determining avian HEV prevalence may also be factors conducive to the observed differences.

Previous studies have indicated that age was not a significant factor during HEV spread [11]. However, in the present study, the seroprevalence in adult pet birds (10.37%) was significantly higher than that in juvenile pet birds (2.07%) (OR = 5.48, 95% CI 1.69–17.76, *P* = 0.002); this result is probably due to the fact that the adult pet birds had more chance to contact with avian HEV compared to juvenile pet birds and increased the risk of infection. The more epidemiological surveys should be conducted to reveal the relationship between age of pet birds and the HEV infection.

In China, a great number of pet birds are raised in a semifree range system, which enhances the opportunity of exposure to food or water contaminated with HEV when they gather together. No statistical difference in HEV seroprevalence was found between male pet birds (9.31%, 39/419, 95% CI 6.53–12.09) and the females (7.52%, 20/226, 95% CI 4.35–10.69) (Table 2), probably because they live in the same environment.

The diagnosis of avian HEV infection in the field should be done minimally by detecting the specific virus RNA from the chicken samples [4]. Avian HEV could either cause HS syndrome alone or play an assistant role coupled with other agents in strengthening clinical symptoms. Despite the fact that the present study reported seropositivity in Eurasian siskin, Oriental skylark, and black-tailed grosbeak, we did not conduct the observation of clinical symptoms in these birds due to limited time. In future studies, it would be interesting to enlarge the variety and number of sampled birds. Furthermore, further studies should be conducted to detect the specific virus RNA in birds sample and examine whether or not avian HEV infection can aggravate clinical symptoms via coinfection experiments with other agents and illuminate the clinical significance of avian HEV infections in China.

## 5. Conclusion

In summary, the present study revealed for the first time the seroprevalence of HEV infection in three species of pet birds in northwest China, which offered the baseline data for taking comprehensive countermeasures and measures for effectively preventing and controlling HEV infection in birds and we believe that this information will be significant for researching the hepatitis E virus (HEV) infection in birds in China.

## Figures and Tables

**Table 1:** Seroprevalence of HEV infection in pet birds in Gansu, northwest China.

Host	Site	Number of tested	Number of positive	Prevalence % (95% CI)	*P* value	OR (95% CI)
Eurasian siskin	Lanzhou	120	18	15.00 (8.61–21.39)		Reference
	Tianshui	120	9	7.50 (2.79–12.21)	0.066	0.46 (0.20–1.07)
	Longnan	120	12	10.00 (4.63–15.37)	0.242	0.63 (0.29–1.37)

Oriental skylark	Lanzhou	169	13	7.69 (3.68–11.71)		Reference
	Tianshui	40	2	5.00 (0.00–11.75)	0.553	0.63 (0.14–2.92)
	Longnan	80	4	5.00 (0.22–9.78)	0.432	0.63 (0.20–2.00)

Black-tailed grosbeak	Tianshui	36	1	2.29 (0.00–8.15)	—	—

**Table 2:** Analysis of the variables associated with HEV seroprevalence in pet birds in northwest China.

Variable	Category	Number of tested	Number of positive	Prevalence % (95% CI)	*P* value	OR (95% CI)
Region	Tianshui	289	12	4.15 (1.85–6.45)		Reference
	Lanzhou	196	31	15.82 (10.71–20.93)	<0.001	4.34 (2.17–8.68)
	Longnan	200	16	8.00 (4.24–11.76)	0.072	2.01 (0.94–4.34)

Gender	Male	419	39	9.31 (6.53–12.09)		Reference
	Female	266	20	7.52 (4.35–10.69)	0.416	0.79 (0.45–1.39)

Species	Black-tailed grosbeak	36	1	2.29 (0.00–8.15)		Reference
	Oriental skylark	289	19	6.57 (3.72–9.43)	0.371	2.38 (0.31–18.29)
	Eurasian siskin	360	39	10.83 (7.62–14.04)	0.126	4.25 (0.57–31.91)

Age	Juvenile	145	3	2.07 (0.00–4.39)		Reference
	Adult	540	56	10.37 (7.80–12.94)	0.002	5.48 (1.69–17.76)

Total		685	59	8.61 (6.51–10.71)		
